# Development and performance assessment of a new opensource Bayesian inference R platform for building energy model calibration

**DOI:** 10.1007/s44245-023-00027-2

**Published:** 2023-10-31

**Authors:** Danlin Hou, Dongxue Zhan, Liangzhu Wang, Ibrahim Galal Hassan, Nurettin Sezer

**Affiliations:** 1 Centre for Zero Energy Building Studies, Department of Building, Civil and Environmental Engineering, Concordia University, 1455 de Maisonneuve Blvd. West, Montreal, QC H3G 1M8 Canada; 2 Mechanical Engineering Program, Texas A&M University at Qatar, Engineering Building, Education City Al Rayyan, P.O. Box 23874, Doha, Qatar

**Keywords:** Calibration, Building energy model, Bayesian inference, Markov Chain Monte Carlo (MCMC), Sensitivity analysis, Uncertainty

## Abstract

Many factors contribute to the inherent uncertainty of energy consumption modeling in buildings. It is essential to perform a calibration and sensitivity analysis in order to manage these uncertainties. Despite the availability of several calibration methods, they are often deterministic and lack quantified uncertainties. Moreover, the selection of parameters in building energy modeling for calibration depends on the user’s experience. Therefore, a more rigorous selection process is required. This study developed a new automated Bayesian Inference calibration platform running as an R package. A sensitivity analysis module and a Bayesian inference module determine the calibration parameters and uncertainties, respectively. The Meta-model module is developed to replace the building energy model for the Markov Chain Monte Carlo process to save computing time. The proposed platform is successfully demonstrated on a synthetic high-rise office building and a real high-rise residential building in a hot and arid climate. The relationship between the number of calibration parameters, calibration performance, and the accuracy of the Meta-model is further discussed. The developed calibration platform in this study proved to have clear advantages over the existing platforms, with the ability to reasonably estimate building energy performance in a short computing time.

## Introduction

International Energy Outlook published by the U.S. Energy Information Administration (EIA) [1] estimates a $\sim$ 50% rise in global energy consumption by 2050 with a 0.8% annual increase in energy-related carbon emissions. Among different sectors, energy consumption by the building sector has increased drastically over the past decades due to the urbanization trend, rapid population growth, as well as increased diversity in building functions. Intensive efforts have been devoted to improving the energy efficiency of buildings, for which computer simulations by Building Energy Models (BEMs) play a crucial role. The accuracy of a BEM could directly determine the quality of different energy-saving measures. There are three possible ways of improving the accuracy of a BEM. The first is providing highly accurate values for the model’s input parameters. However, it is limited by the availability and quality of the data. The second is improving the simulation algorithm by carefully analyzing the model and finding possible ways to optimize its performance. However, it often requires extensive time, resources, and technical expertise. The third is model calibration, which may be the most effective way of improving model accuracy. Based on the optimization/estimation methods available, the optimum combination of model parameters with specific model inputs is identified to align the simulation results with actual measurements.

Model calibration can be conducted manually or automatedly [2]. A manual calibration approach relies on a user's expertise in building science, computer simulation, and his/her knowledge about the features of the target building. In manual calibration, several key parameters are manually selected and adjusted to align the simulation results to the measurements. Thus, manual calibration is a time-consuming, labor-intensive, and expensive process [3, 4]. Besides, the manually calibrated model may often be questionable due to the user's limited expertise and the calibration problem's complexity.

Automated calibration is often preferred when computer resources and tools are available to support the automated process [5]. It is a non-user-driven and mathematics-based process to match simulation results with measured data [6]. With the mathematical/statistical methods coded in a computer program, the calibration activity can be iterated automatically for a large batch of simulations with various combinations of parameters. The automated search process is considered complete when the calibration error is less than a threshold criterion or the calibration activity runs longer than the predefined period. The group of input parameters of a specific simulation with the lowest error is selected as the calibration results.

Currently, several calibration platforms have been developed and released. Some of them were embedded within a building energy simulation software, such as the DesignBuilder Optimization module [7], the Parametric Analysis Tool for OpenStudio [8], and the TESS Optimization Library of TRNSYS [9]. Others were designed as software to work with certain building energy models [10–12]. For example, the US Lawrence Berkeley National Laboratory developed a web-based platform for California's small-to-medium office and retail buildings [13]. They also extended the platform for building energy model calibration from single building to district/city scale [14, 15]. For the platform of Autotune, supercomputer-assisted generation of machine learning agents was employed to calibrate building energy models [16]. The Hydro-Québec Research Institute in Canada created ExCalibBEM using GenOpt, an optimization engine, and building performance simulation tools to process the calibration [17]. Multi-Objective Building Optimization Tool (MOBO) [18] focuses on IDA-ICE and TRNSYS models' calibration using optimization methods. However, the calibration results of these platforms are often deterministic, and no uncertainty is considered. The calibration parameters can be far off from their original value when the quality/quantity of the calibration data is limited. Considering the variety of uncertainties, a BEM with probability outputs seems more intuitively reasonable. In addition, to employ the platform for calibration, users often need to select calibration parameters without a guide. Therefore, the selection has to rely on the rule of thumb and subjective decisions, which subsequently affects the final calibration results.

Bayesian Inference is a useful method that absorbs information extracted from observations to update the information we don't know but want to know. Uncertainties can be considered in the inference process since it uses probability models. As an effective tool to interpret and quantify these uncertainties, Bayesian Inference has gained a broad interest. Bayesian Inference is derived from the Bayes' theorem proposed in 1763 [19] and gained momentum with the development of Markov Chain Monte Carlo and modern computers. Its application became popular after the study of Kennedy and O'Hagan on the Bayesian calibration of computer models [20]. One of the earliest applications of Bayesian inference in building energy modeling was presented by Heo [21] on building retrofit risk estimation. Since then, Bayesian inference has gained researchers' great interest in building energy fields and has been applied to other research fields, such as prediction of stock energy consumption [22–25], retrofit analysis [21, 26–28], calibrating unknown parameters [29–31], and identifying informative energy data [28, 32]. For more information, please refer to our previous published review paper regarding the applications of Bayesian inference for BEM calibration and the proposed systematic Bayesian calibration procedure [33].

Various studies presented Bayesian Inference calibration procedures on a single building or urban scale [34]. However, it remains a challenge for new users to understand the underlying theory, methodology, and implementation of Bayesian Inference. Some researchers shared their Bayesian calibration code to facilitate the application for new users. However, modifying the code developed in R [35] and Stan [36] is still challenging for readers who do not have in-depth expertise in these programming languages [37].

During the calibration process, identifying all parameters of a detailed building energy model is not possible [38]. Alternatively, a solution set of model parameters identified for calibration can be used. Sensitivity analysis is a scientific approach to investigating how various input uncertainties can apportion the output's uncertainty. Therefore, it can identify the input variables that have the most significant impact on the output. Sensitivity analysis cannot only be applied to building layout design and building envelope evaluation but also be applied to model calibration as an essential step for choosing the critical parameters for calibration. However, it has not been implemented in most available calibration platforms yet.

Despite the availability of various calibration platforms, a new calibration platform is still necessary to ensure an accurate estimation of the energy performance of all building types in a reasonable computing time by combining parametric simulation, sensitivity analysis, and Meta-model development in one program. This paper uses previous knowledge of building energy model calibration (e.g., [32, 37, 39, 40]) to develop a new calibration platform. A package including parametric simulation, sensitivity analysis, Meta-model development, and Markov Chain Monte Carlo (MCMC) was developed on a single language programming platform, R.

The summary of the characteristics of the new calibration platform developed in this paper against the currently available calibration platforms is summarized in Table 1. Several platforms are commercially available, especially those for BEMs developed in TRNSYS, such as Multiopt2 and TRYOPT. Some platforms lack parallel computing capability, leading to a long calibration computing time. A significant concern of users is the platform's ability to deal with discrete and continuous variables simultaneously. Some platforms can focus on only one or two building types. For instance, BEopt [10] is developed for residential buildings, while commercial Building Energy Saver (CBES) [13] works for small/medium office and retail buildings. On the other hand, Opt-E-Plus [12] is limited to commercial ones only. Only a few of the platforms are equipped with sensitivity analysis, whose results can be used for the determination of calibration parameters. Furthermore, none of the currently available platforms can develop Meta-models used in the calibration process instead of the original BEM to reduce computing time.

**Table 1:** Characteristics of the available building energy model calibration platforms and the proposed platform**

NO	Tool	Freeware	Parallel computing	Variables (discrete & continuous)	Sensitivity analysis	Meta-model development	Uncertainty prediction	All buildings	Free BES	Calibration method
1	Autotune	√	–	√	x	x	x	√	√	Machine learning
2	BEopt	√	x	x	x	x	x	x	√	Optimization
3	City Building Energy Saver (CityBES)	√	–	–	x	x	x	√	√	Pattern recognition
4	Commercial Building Energy Saver (CBES)	√	–	–	x	x	x	x	√	Hierarchy calibration
5	DesignBuilder Optimization module	x	–	√	√	x	√	√	x	Optimization
6	ExCalibBEM	√	–	√	x	x	x	√	√	Optimization (GenOpt)
7	jEPlus + EA	x	x	x	x	x	x	√	√/ x*	Optimization (Enhanced NSGA2-based algorithm)
8	Multi-Objective Building Optimization Tool (MOBO)	√	√	√	x	x	x	√	x	Optimization
9	Multiopt2	x	√	√	x	x	x	√	x	Optimization
10	Opt-E-Plus	√	x	x	x	x	x	x	√	Optimization
11	Parametric Analysis Tool (PAT)	√	–	√	x	x	x	√	√	Optimization
12	TESS Optimization Library (TRYOPT)	x	√	√	x	x	x	√	x	Optimization (GenOpt)
13	This study	√	√	√	√	√	√	√	√	Bayesian inference

√/ x* indicates that the answer is "yes" for some BESs and "no" for other BESs

^**^ https://github.com/CUBELeonwang/Bayesian

An innovative feature of this work is the development of a novel automated Bayesian calibration system, which is implemented as an R package and facilitates a seamless calibration process that begins with the visualization of measurement data. A key feature of the system is its ability to accommodate all BEMs in EnergyPlus. Our platform also includes a sensitivity analysis module, which can be used to determine the parameters that need to be calibrated, based on the characteristics of the building. Parallel computing and the development of meta-models significantly reduce the time required for calibration calculations. Moreover, our platform utilizes Bayesian Inference to ensure that the results are closely aligned with the actual data. Consequently, this attribute contributes to the reliability of results and minimizes deviations. In addition, we exclusively use the R programming language for data analysis. In contrast, ref [26] uses the Stan programming language, a probabilities programming language that may pose coding challenges for readers without in-depth expertise in these languages. For the R code for the proposed platform in this paper, please refer to: https://github.com/CUBELeonwang/Bayesian.

The remainder of this paper is organized as follows. The Bayesian Inference calibration procedure for BEM and its implementation are described in Sect. 2. Two case studies, an office building and a residential building in a hot and arid climate, are provided in Sect. 3 and Sect. 4, respectively. Calibration parameter numbers, Meta-model accuracy, calibration performance, and computational cost are discussed in Sect. 5. Finally, the conclusions of the study and future research directions are provided.

## Methodology

**Fig. 1:**
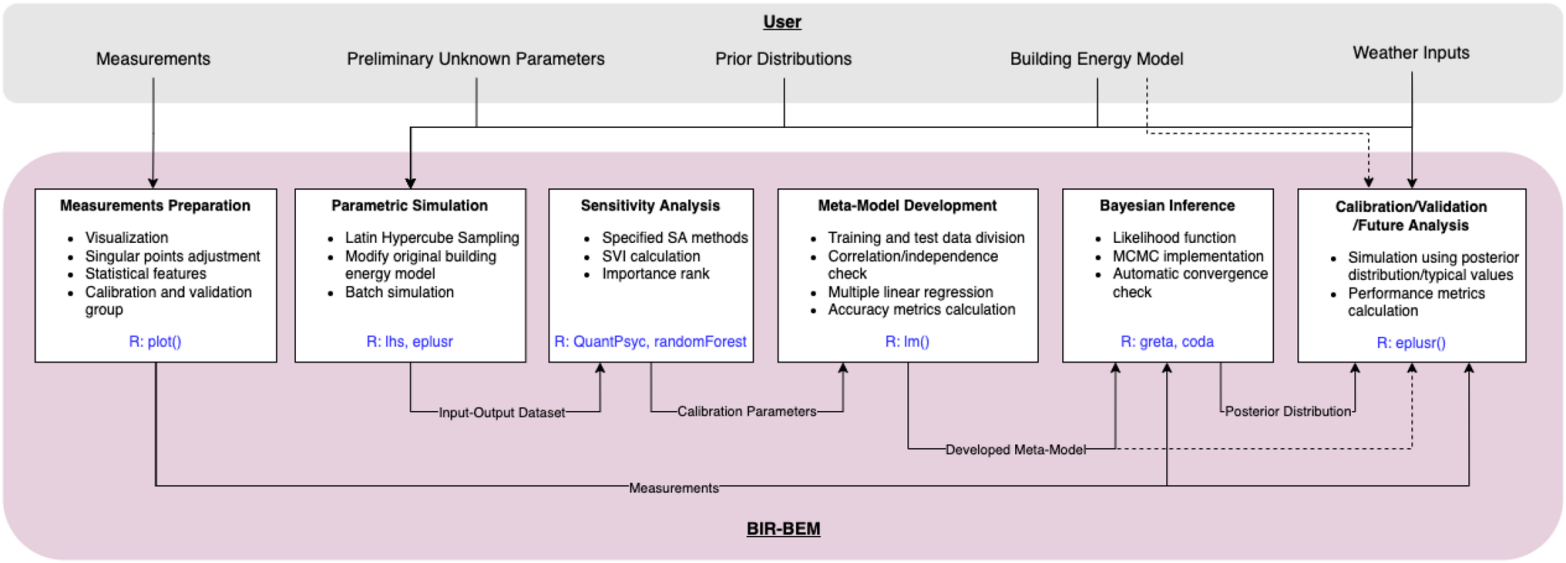
A schematic workflow for the calibration procedure of the proposed platform. Words with brackets mean R base functions, while others represent the R packages

One of the key aspects of this methodology is using the R package to demonstrate the whole calibration process step by step, so that the readers will be able to follow along as the process progresses. The calibration procedure of the proposed platform is illustrated in Fig. 1. The information that users should provide is listed in the grey rectangle. Modules with identified capabilities and inputs/outputs are shown in the pink area. A solid line indicates necessary information, while a dashed line means optional. The whole process is created using a single programming environment, R [41, 42], a widely used statistical programming language. The implementation of each step is highlighted in blue color in Fig. 1. In addition, the methodology section encompasses the method of sensitivity analysis, meta-model development, Bayesian inference calibration and performance evaluation metrics.

### Measurement preparation

The first step of using the platform is measurement preparation. Modelers should be familiar with basic information about the energy data, such as data type and measurement time resolution. Data points that stood out from the rest of the dataset were considered singular points and have been removed based on visual observation using the R function “plot()”. These points can later be removed or adjusted if needed. Features of the measurement data, such as data number, mean, mode, and median values, can be summarized and output. Also, the data can be grouped into two sets for calibration and validation. By default, the first 2/3 of measurements are used for calibration and the reminders for validation. Users can change the separation ratio. The output of this step is employed in the Bayesian Inference process and calibration/validation performance assessment.

### Building energy model and unknown parameters

Users should provide a BEM created for the target building based on collected/audited building information. Several simulation tools are available for the development of a building energy model, including but not limited to DOE-2 [43], EnergyPlus [44], TRNSYS [45], and ESP-r [46]. User-developed models can also be employed to create building energy models. According to previous studies, different BEMs will not significantly impact the Bayesian Inference calibration performance [29, 47]. However, the feasibility of the automatic parametric simulation and input–output dataset extraction should be considered. EnergyPlus is one of the most popular BEM tools due to its flexibility and accuracy in modeling a building and its systems. An R package, "eplusr", is available for EnergyPlus that is capable of programmatic navigation, parameter modification, parametric simulations, and retrievals of outputs [48]. Thus, this study selected EnergyPlus as the building energy modeling tool.

Preliminary unknown important model parameters should be determined based on users' experience or from the literature. The distributions and ranges of these parameters should be set according to building design codes/standards to ensure that the sensitivity analysis results in the following step are valid and useful.

### Parametric simulation

As discussed in the previous section, users should define the prior distributions and ranges of preliminary unknown parameters in practice. Then a specific sampling method, such as the Latin Hypercube sampling (LHS) method [49], is applied for sampling the selected parameters from the prior distributions. The sampling can be achieved by using the R package "lhs." The generated combination groups of samples are used as the inputs of the BEM to conduct the parametric simulation. The parametric simulation can be referred to as the "uncertainty propagation" and is performed automatically using the R "eplusr" package, which can also fulfill the input–output dataset extraction. The LHS with output simulation results forms an input–output dataset which is used for sensitivity analysis and Meta-model development.

### Sensitivity analysis

Based on the input–output dataset, the dominant model parameters, strongly affecting the outputs, can be identified using sensitivity analysis. Tian summarized various sensitivity analysis methods, such as Morris, Lasso, Standard Regression Coefficient (SRC), and Random Forest Variable Importance (RFVI), which have been used in the Bayesian Inference framework [50]. However, the results of the importance rank may vary with different combinations of sensitivity methods and outputs depending on the variety of fundamental algorithms and conditions for each sensitivity analysis method [51]. Lim and Zhai proposed a new sensitivity analysis index, Sensitivity Value Index (SVI), which avoids any inconsistencies in results by taking into account the differences in sensitivity analysis methods and target outputs [40]. Equation 1 shows how SVI is applied to recognizing and comparing the importance rankings from different sensitivity analysis methods through the normalization and aggregation process. In this study, three sensitivity analyses (SRC, RFVI, and T-value) are applied, which can be conducted using the R package "QuantPsyc" and "randomForest".

1 $$ SVI \left( \% \right) = \mathop \sum \limits_{l = 1}^{m} \frac{{\mathop \sum \nolimits_{j = 1}^{k} \left( {\frac{{v_{i,j} }}{{\mathop \sum \nolimits_{i = 1}^{n} \left| {v_{i,j} } \right|}}} \right)}}{{m \cdot k^{2} }} \times 100 $$

where $v$ is the value from a particular sensitivity analysis method, $i$ is a model parameter, $n$ is the total number of the parameters, $j$ is a sensitivity method, $k$ is the total number of sensitivity methods (k = 3 in this study), $l$ is a specific target output, and $m$ is the total number of target outputs.

### Meta-model development

A software tool is a simulator that replicates actual phenomena. A Meta-model (also called surrogate model) is a simplified representation or approximation (i.e., an emulator) of the simulator for saving computing time. Generally, several models can be used as a Meta-model in Bayesian Inference to replace original BEMs, such as Multiple Linear Regression (MLR), Neural Network (NN), Support Vector Machine (SVM), Multivariate Adaptive Regression Splines (MARS), and Gaussian Process Emulator (GPE). According to a study by Lim and Zhai [40], when MLR is employed to represent the original EnergyPlus simulation for a case study of DOE reference medium office building, the computing time decreased from 70 days to 2.2 min for an MCMC process with 100,000 iterations using a computer with Intel Core CPU (i7-4790 3.6 GHz) and 12GB RAM. Besides fast computing with the MLR model, the calibration performance was also in an acceptable range. Therefore, in this study, MLR is selected as the Meta-model in Bayesian Inference. The simulated input–output dataset generated from the parametric simulation is divided into two parts for the MLR development: the first 2/3 is for training, while the rest is for its accuracy test.

R^2^ and Residual Standard Error (RSE) are used to define the performance of the Meta-model as represented in the following equations.

2 $$ R^{2} = 1 - \frac{{\mathop \sum \nolimits_{i = 1}^{n} \left( {\hat{y}_{i} - \overline{y}} \right)^{2} }}{{\mathop \sum \nolimits_{i = 1}^{n} \left( {y_{i} - \overline{y}} \right)^{2} }} $$

3 $$ RSE = \sqrt {\frac{{\mathop \sum \nolimits_{i = 1}^{n} \left( {y_{i} - \hat{y}_{i} } \right)^{2} }}{n - 2}} $$

where ${\widehat{y}}_{i}$ is a predicted variable value for period $i$, ${y}_{i}$ is an observed value for period $i$, $\overline{y }$ is the mean of the observed value, and $n$ is the sample size.

### Bayesian inference calibration

As the footstone of all Bayesian statistics, Bayes' theorem was first proposed by Reverend Thomas Bayes in his doctoral dissertation [19], which is described in the Eq. (4).

4 $$ Posterior = \frac{Probability\,of\,the\,data\,times\times\, Prior}{{Average\,probability\,of\,the\,data}} $$

The probability of an event is inferred based on the prior knowledge of conditions that might be related to the event. Bayesian Inference is one application of Bayes' theorem and can be expressed in the Eq. (5).

5 $$ p\left( {\theta {|}y} \right) = \frac{{p\left( {y{|}\theta } \right) \cdot p\left( \theta \right)}}{p\left( y \right)} \propto p\left( {y{|}\theta } \right) \cdot p\left( \theta \right) $$

where $p\left(\theta |y\right)$ is the posterior distribution of the calibration parameters *θ* based on the known observations *y*, and $p\left(y|\theta \right)$ is the likelihood function of observations conditional on the unknown calibration parameters, which can be obtained from information from measurements. Generally, a Gaussian distribution is assigned according to the building's monthly energy consumption profile characteristics. $p(\theta )$ is the prior distribution of the unknown parameters that users should define according to requirements of building codes/standards and engineering experience, and $p(y)$ is the probability of the observations to normalize $p\left(y|\theta \right)$. Therefore, the posterior probability is proportional to the product of the prior probability and the likelihood.

In reality, Bayesian Inference cannot provide analytical solutions to all kinds of problems since calculating the likelihood integrals can be computationally intensive or even mathematically intractable in some instances. MCMC is a versatile approach to solving the parameter estimation problem with two components. One is the well-known Monte Carlo method to solve statistically challenging problems by random samplings. The other is the Markov Chain method for solving a sequence of possible events, in which the probability of each event depends only on the state attained in the previous event. By integrating MCMC and Bayesian Inference, posterior distribution can be estimated efficiently. Hamiltonian Monte Carlo (HMC) is an efficient MCMC algorithm. It utilizes first-order gradient information to determine how samplers should move to the target distribution [52]. This moving approach can guarantee it converges to the target distribution more quickly, especially for a complicated high-dimensional problem. It is noted that during the MCMC process, the MLR model is employed instead of the original BEM to reduce computing costs. The output of the MLR model is used to estimate the mean value of the Gaussian distribution, which is defined as the likelihood function. Convergence criteria should be satisfied to ensure the MCMC samplings are generated from the posterior distribution to guarantee that the results are steady and meaningful. R libraries of "greta" and "coda" are employed in this step due to the former's plot function to visualize and check the relationship between the unknown parameters and the latter's capability of simply convergence check.

### Calibration and validation performance

By using mean values of the posterior distributions to set the original BEM model, the calibration and validation performance can be assessed using two criteria. One is the error (the difference ratio between the calibration parameter's estimated value and its actual value) and the other is Coefficient of Variation with a Root-Mean-Square Error (CVRMSE), which is calculated by normalizing the Root Mean Squared Error (RMSE) with the mean of the data and provides a unit-less percentage value (Eq. 6). ASHRAE Guideline 14 recommends the use of CVRMSE as a statistical metric for calibrating energy models [53]. In this way, the Bayesian Inference-based stochastic BEM, generated based on the samplings from posterior distributions with the original BEM or the developed Meta-model, can be further analyzed.

6 $$ CVRMSE = \frac{{\sqrt {\frac{{\mathop \sum \nolimits_{{i = 1}}^{n} \left( {y_{i}  - \hat{y}_{i} } \right)^{2} }}{n}} }}{{\bar{y}}} $$

where ${y}_{i}$ indicate measured values, ${\widehat{y}}_{i}$ indicate modelled values, and $\overline{y }$ is the mean of the measured dataset.

## Case 1: a synthetic office building

This section describes the step-by-step implementation of the calibration platform on a synthetic office building. First, a building model was created, and the primary model's unknown parameters were selected to conduct the sensitivity analysis. Based on the sensitivity analysis results, the first five critical unknown parameters were injected into the MLR model and then calibrated to evaluate the Bayesian calibration performance in recovering the original model.

### Building energy model

**Fig. 2:**
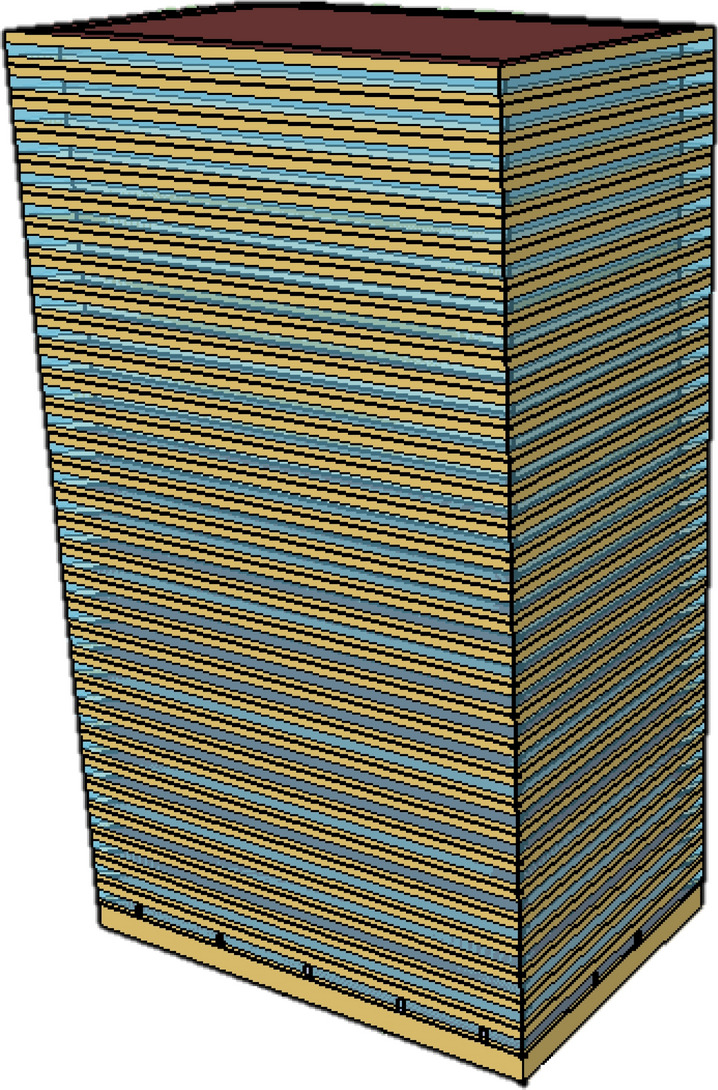
EnergyPlus model of the synthetic office building

This study developed a general office building model based on the online information collected from more than 70 office buildings in Doha, Qatar, including their floor number and total floor area [54]. The model is for an office building with a total floor area of 3504 m^2^, 31 floors, and a basement floor. Each floor is divided into five conditioned zones (four perimeters, one core) as shown in Fig. 2. Windows are on all four facades and the window-to-wall ratio is 40%. A fan-powered variable-air-volume system provides air conditioning in the five zones. According to the literature, a stand-alone cooling system is typically applied to BEMs in Qatar to supply over 80% of the country’s building cooling demand [55]. A summary of the building characteristics is provided in Table 2. A general building energy model is selected instead of an actual building because it is impractical to obtain "true" values of model parameters for a real building. Since this study aims to estimate the Bayesian Inference calibration performance, a baseline (reference values of model parameters) should be available for comparison.

**Table 2:** Main features of the office building

Component	Parameter	Value	Unit
Envelop	Floor area	3504 (73 $\times 48$)	m^2^
	Number of floors	31 (+ 1 basement floor)	–
	Window-to-wall ratio	40% of above-grade gross walls	–
	Thermal zoning	Four perimeter zones and one core zone on each floor	–
Internal heat gains	Lighting power density	See Table 3	W/m^2^
	Equipment power density	See Table 3	W/m^2^
	Occupancy density	See Table 3	m^2^/person
	Hourly schedules for heating and cooling setpoint, occupants, lights, and equipment	The default setting of the DOE reference office building	–
HVAC system	System type	VAV	
	Heating type	Gas boiler	
	Cooling type	Electric chiller	

### Calibration parameters

The Bayesian calibration platform requires a list of calibration parameters, which are altered to calibrate the model according to the measured data. The user should define a primary potential calibration parameter list based on his/her knowledge and local building standards/codes. The primary unknown parameters are stored in a *.csv file, which allows a user to define: a class, object, and field to identify any parameter in an EnergyPlus input file uniquely. Distributions of uncertainties (e.g., uniform, normal, and triangular) with their key factors can also be specified in the same file. The parametric simulation will be conducted, and the sensitivity analysis results will be output in another *.csv file.

According to ASHRAE Standard 169 [56], hot/arid regions are defined as climate zone 0B. For Doha, a coastal city in Qatar, its summer is long, hot, and humid from May to September, while winter (December, January, and February) is mild, and spring (March and April) and autumn (October and November) are warm. Due to a long period of high temperatures, building thermal insulation is required more strictly in such a hot/arid region. In our study, 14 unknown model parameters (Table 3) selected from previous studies are used for sensitivity analysis. The ranges for these parameters have been defined in Qatari building design codes and their dependent international standards, such as ASHRAE standards [57–60]. Some parameters are adjusted to make the building more energy-saving, such as a higher cooling setpoint (typically, it is between 18 and 20 $^\circ{\rm C}$ [61]), especially during the unoccupied period, to respond to the sustainable development requirements of Qatar. Initially, each parameter is set with a uniform distribution for its range. The uncertainties of occupant behavior are not investigated in much detail due to their complexity.

**Table 3:** The list of model input parameters and their ranges

Parameter	Abbreviation	Range	Unit
Roof insulation U-Value	RINU	0.01–0.25	W/m^2^ $\bullet$ K
Wall U-Value	WALU	0.01–0.30	W/m^2^ $\bullet$ K
Floor U-Value	FLOU	0.50–1.80	W/m^2^ $\bullet$ K
Window U-Value	WINU	0.01–1.80	W/m^2^ $\bullet$ K
Window SHGC	SHGC	0.0–0.2	–
Equipment power density	EPD	11.0–15.0	W/ m^2^
Lighting power density	LPD	5.0–9.0	W/ m^2^
Occupancy	OCC	15.0–25.0	m^2^/person
Infiltration	INF	0–2.0E-3	m^3^/s·m^2^
Ventilation	VEN	4.7E-4–2.5E-3	m^3^/s·m^2^
Cooling setpoint	CSP	Occupied: 22.5–25.5; Unoccupied: 25.5–28.5	$\mathrm{^\circ{\rm C} }$
Heating setpoint	HSP	Occupied: 18.0–22.5; Unoccupied: 15.0–18.0	$\mathrm{^\circ{\rm C} }$
Chiller COP	COP	3.3–6.0	–
Boiler efficiency	EFF	0.80–0.98	–

Matala's recommendation for the sampling size of the LHS advised a sample size of 50 to ensure that the sample's distribution was not significantly different from the original distribution [62]. For the sensitivity analysis in this study, we conducted 700 parametric simulations for 14 parameters. According to the sensitivity analysis results, the first five significant parameters are selected to develop and calibrate the MLR model during the MCMC process. Although the default value for the significant parameters is set as five, users can determine the number of calibration parameters based on their experience or calibration accuracy requirement.

### Calibration data

Calibration data can be provided in a *.csv file with two columns. The first column serves as the date/time, whereas the other column contains measured data from that timestep. The header of the second column must correspond to an EnergyPlus output. For demonstration purposes, in this study, a selected dataset from the testing trunk (i.e., the first test sampler) is used as the target building. The testing trunk refers to the partial dataset obtained from the parametric simulation, which was used to test the developed MLR Meta-model. The sampler's input parameters and output energy consumption are considered reference model parameters and measurement values. The monthly electricity consumption was regarded as the “measured” data. Most studies used monthly data, especially for the Bayesian Inference calibration process [33]. However, higher-resolution data is preferred when it is available.

### Running calibration

First, an MLR Meta-model will be developed whose independent variables are calibration parameters obtained from the sensitivity analysis results. The MLR model will be employed during the MCMC process. MLR was selected to represent the relationship between monthly Energy Use Intensity (EUI) and to determine calibration model parameters because of its robustness and low risk of overfitting with many variations [63]. Besides, Hamiltonian Monte Carlo (HMC) sampling method [64] was used for the MCMC. Two thousand steps of the HMC algorithms were explored on each of 4 separate chains to make a total of 8000 samplers. One thousand samples were used during the "warming up" stage to move chains toward the highest density area and tune sampler hyperparameters.

### Results

#### Energy consumption

**Fig. 3:**
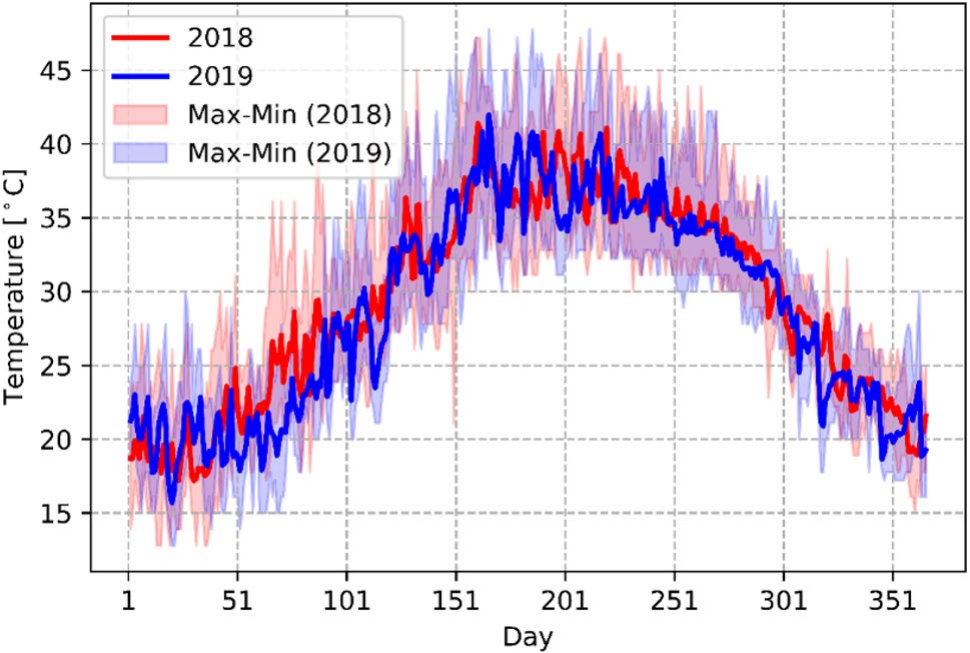
Hourly outdoor air dry-bulb temperature of Doha, Qatar

**Fig. 4:**
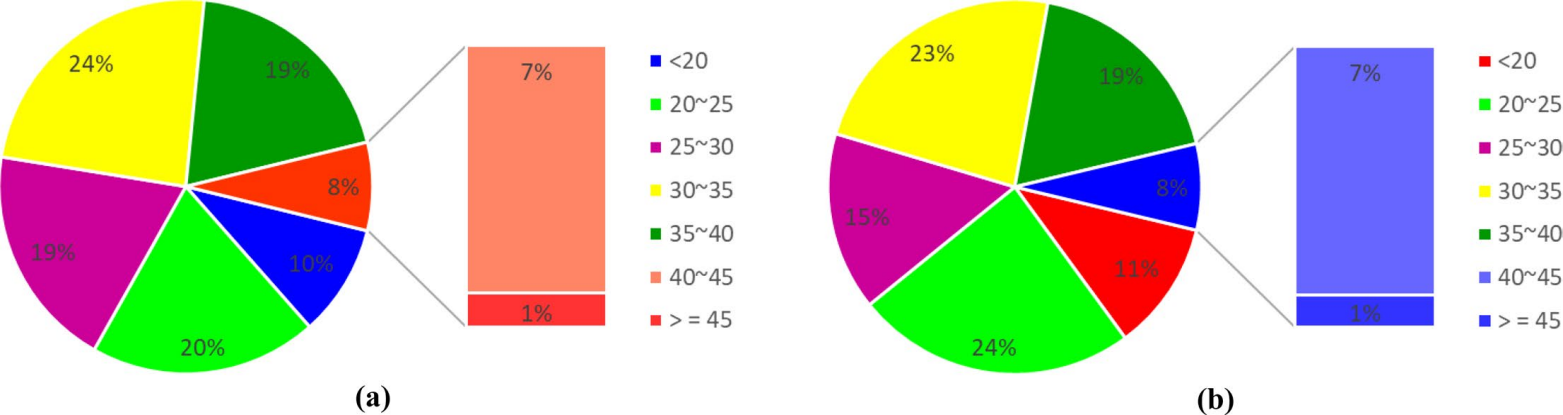
Percentage of hours of outdoor air dry-bulb temperature ($^\circ{\rm C}$) in Doha, Qatar, in **a** 2018 and **b** 2019

Instead of using a Typical Meteorological Year (TMY) file for EnergyPlus simulation, measured hourly outdoor air dry-bulb temperatures in 2018 and 2019 were applied to the calibration and validation, respectively (Fig. 3). In this way, the calibrated model can be more robust by considering the weather impacts from different sources. Parametric simulations were conducted using 2018 weather data to generate an input–output dataset for sensitivity analysis and MLR model development. Hourly percentage of outdoor air dry-bulb temperature ranges is presented in Fig. 4. The peak temperatures of 47.2 $^\circ{\rm C}$ and 47.7 $^\circ{\rm C}$ were observed in 2018 and 2019, respectively. During the year, around 8% of the hourly temperatures exceed 40 $^\circ{\rm C}$ while 30% is below 25 $^\circ{\rm C}$.

**Fig. 5:**
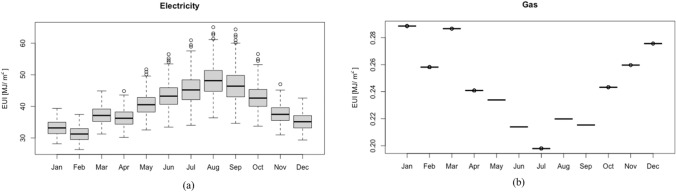
Monthly energy use intensity of 700 samplers in 2018. **a** Electricity, **b** Gas

The EUI (MJ/m^2^) for electricity and gas were used as energy performance indicators. Figure 5 shows the monthly EUI for the 700 samplers and their average values. Electricity usage is dominant for the office building due to the enormous electric office supplies and long-term cooling model operation with electric chillers. The gas fluctuation caused by the uncertainty propagation of heating setpoint and boiler efficiency is unnoticeable since most of the gas consumption is used for domestic hot water service, and the heating hours are rare in hot/arid areas.

#### Sensitivity analysis results

The SVI's comprehensive results are described based on three sensitivity analysis methods, SRC, Random Forest variable importance, and T-value. Table 4 shows the sensitivity analysis results with the importance ranks for the annual total energy consumption. The most critical parameter for the annual total energy consumption is ranked 1. The most dominant parameters for the office building in hot/arid areas are identified as EPD, COP, INF, CSP, and SHGC. Equipment consumes significant electricity in an office building, and COP and cooling setpoint are two key parameters of an air conditioning system operating for long periods during the year. Fresh air infiltration and solar heat gain also account for a large portion of the cooling load. According to ASHRAE Standards 90.1-2019 [58], the requirements for the envelopes of buildings in climate zone 0B are pretty high, as shown by low U-Values, e.g., the wall U-Values are almost half of those in a warmer climate zone 3. As a result, the envelop parameters are not crucial due to their insignificant contribution to the total cooling energy demand. Since the heating period in Doha is very short, the heating setpoint and boiler efficiency are the least important parameters.

**Table 4:** Sensitivity and importance rank of model input parameters

Parameter	Short name	Sensitivity analysis method			SVI	Ranking
		SRC	Random forest	T-value		
Equipment power density	EPD	0.7	145.6	155.0	33.6	1
Chiller COP	COP	−0.6	119.6	−129.1	27.8	2
Infiltration	INF	0.3	59.5	70.0	14.6	3
Cooling setpoint	CSP	−0.2	21.1	−40.6	7.3	4
Window SHGC	SHGC	0.1	16.1	33.8	6.0	5
Occupancy	OCC	−0.1	14.1	−31.5	5.5	6
Ventilation	VEN	0.1	5.0	17.5	2.8	7
Lighting power density	LPD	0.2E−1	−1.7	5.3	0.9	8
Wall U-Value	WALU	0.1E−1	0.3	2.9	0.4	9
Roof insulation U-Value	RINU	0.6E−2	1.6	1.4	0.3	10
Floor U-Value	FLOU	0.2E−2	−2.1	−0.5	0.3	11
Boiler efficiency	EFF	6.5E−3	0.5	1.5	0.2	12
Window U-Value	WINU	5.8E−4	−1.6	0.1	0.2	13
Heating setpoint	HSP	5.7E−4	0.6	0.1	0.1	14

**Fig. 6:**
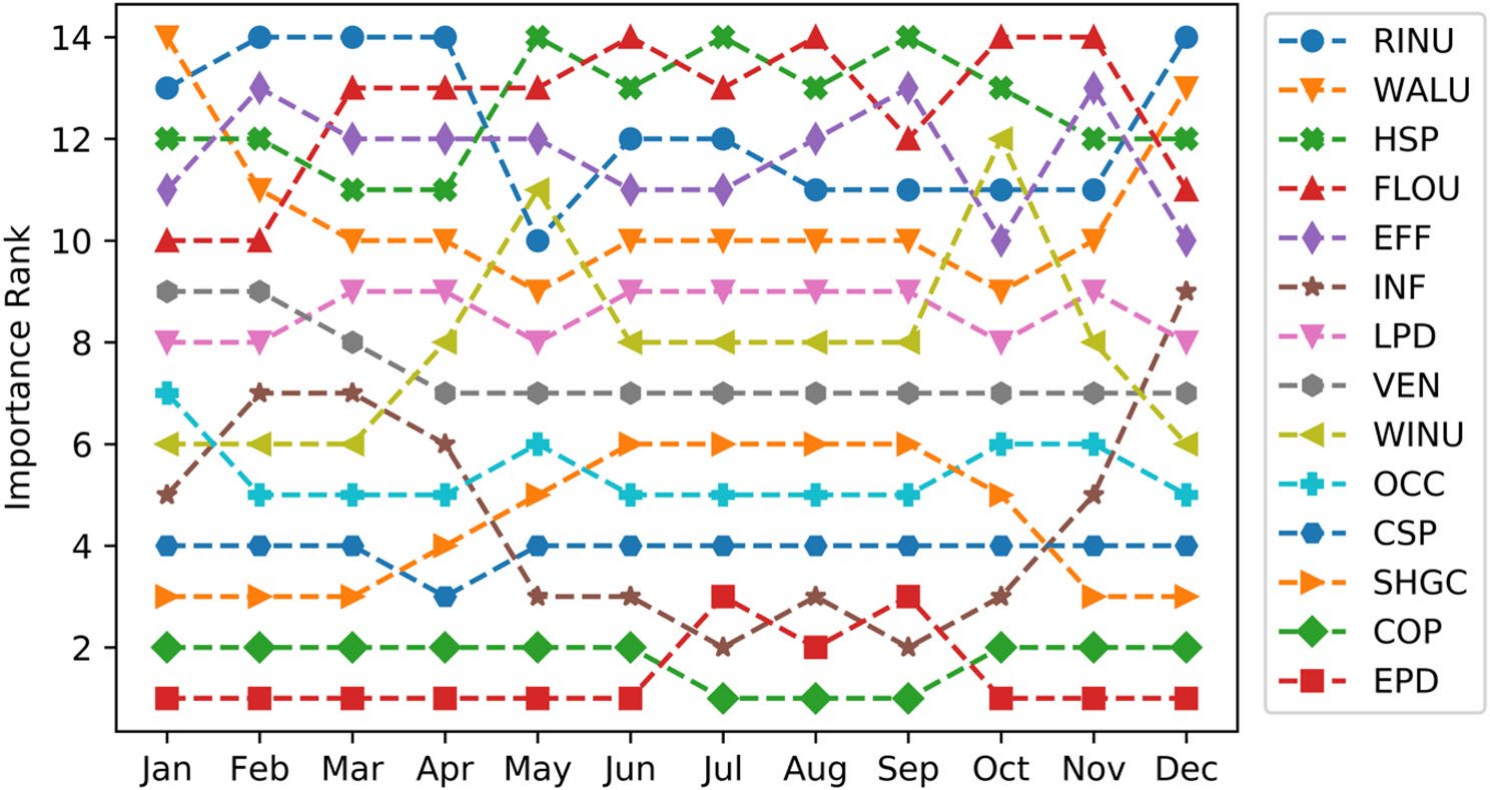
Importance rank of model input parameters

To further explore the importance ranking based on monthly total energy consumption and to observe if it is consistent with annual total energy consumption results, the sensitivity analysis process was repeated for each month. The results are summarized in Fig. 6. Owing to the weather characteristic of Doha, Qatar, where summer is scorching and lasts almost 6 months and the other three seasons are warm and mild, the importance rank results of most parameters are relatively stable through the months of a year. For most parameters, the result trends are similar to the annual total energy consumption. For the INF parameter, the importance rank varied significantly in different months. From May to October, the importance rank is lower, indicating its high impact on building energy consumption, while the significance becomes weaker for other months. It can be explained by the variation in the monthly outdoor air temperature. During summer, high cooling loads are demanded due to the unbearable outdoor air temperature. During other seasons, especially in winter, the outdoor air temperature is mild, and the cooling energy consumption due to the infiltration load reduces.

#### Regression analysis

**Fig. 7:**
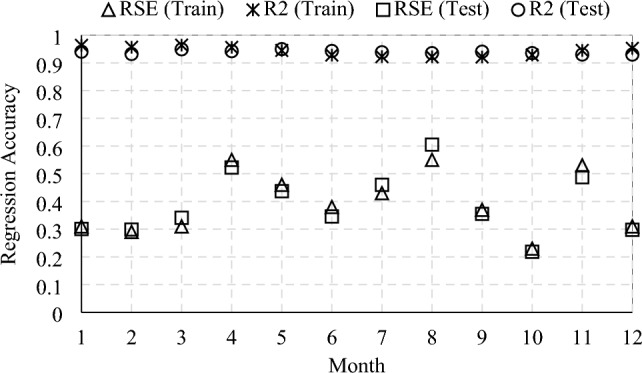
Results of regression analysis for case 1

The results of the five calibration parameters are selected since the optimal number of calibration parameters is identified as five, as discussed in Sect. 5. Besides, the MLR model was chosen as the Meta-model to replace the original EnergyPlus model. Generally, BEMs have complex and nonlinear characteristics, so using a linear model can yield significant errors. However, undeniably, building energy consumption is highly correlated to weather data. When MLR is applied for regression of monthly energy consumption and dominant model parameters, the training and testing accuracy is acceptable, as shown in Fig. 7. The average monthly R^2^ value of both the training set and testing set is 0.94, which is similar to a previous study by Lim and Zhai [32]. Further, the residual standard error of training and test datasets is 0.39.

#### Bayesian inference calibration

##### Convergence

**Fig. 8:**
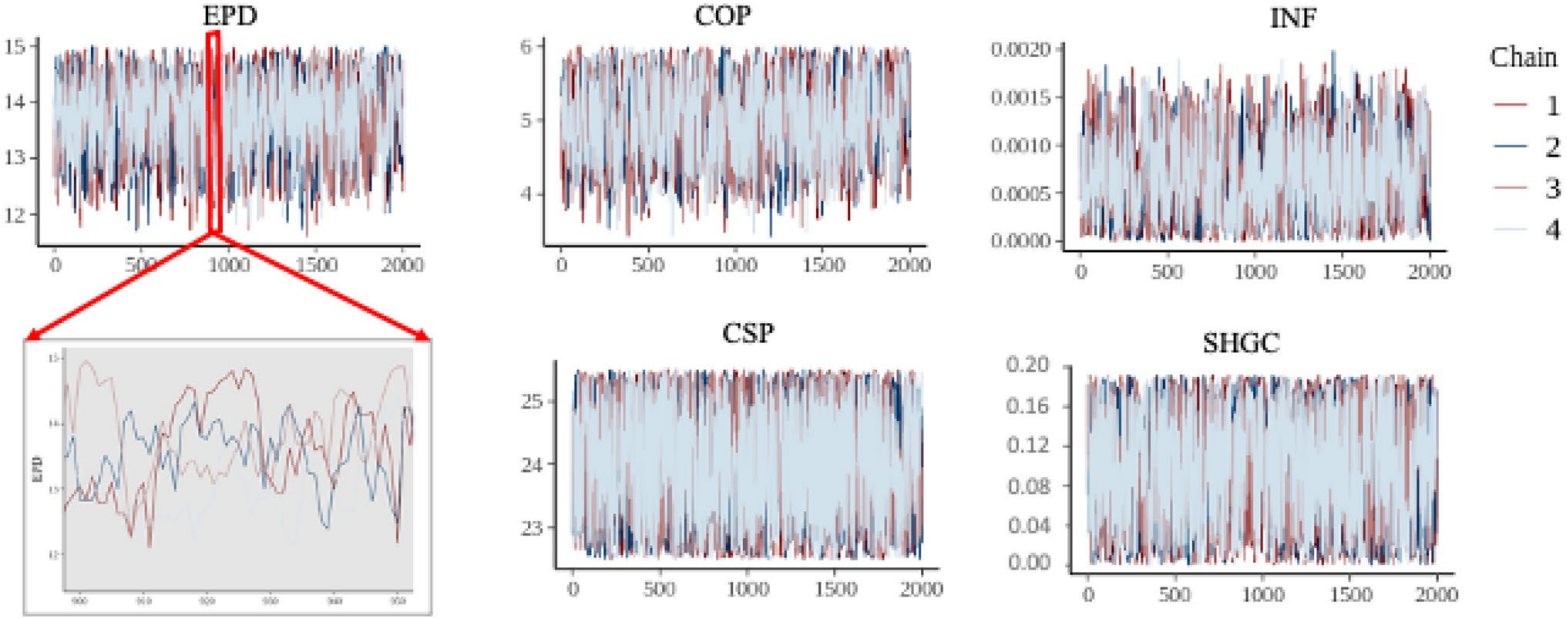
Trace plot of the calibration parameters: entire iterations of the fourth chain with detailed trace plot of equipment power density trace

Trace plots and Gelman-Rubin statistics were applied to diagnose the convergence achievement for the posterior distribution. A trace plot plots the samplers in sequential order, joined by a line, and it is the first and the best way to diagnose common problems for an analyst. Figure 8a shows the sample trace of five calibration model parameters. For each parameter, the chains are mixed well to be stationary and convergent, which indicates that the MCMC posterior distribution reached the convergence. A detailed trace plot of EPD is included in the figure as well.

**Fig. 9:**
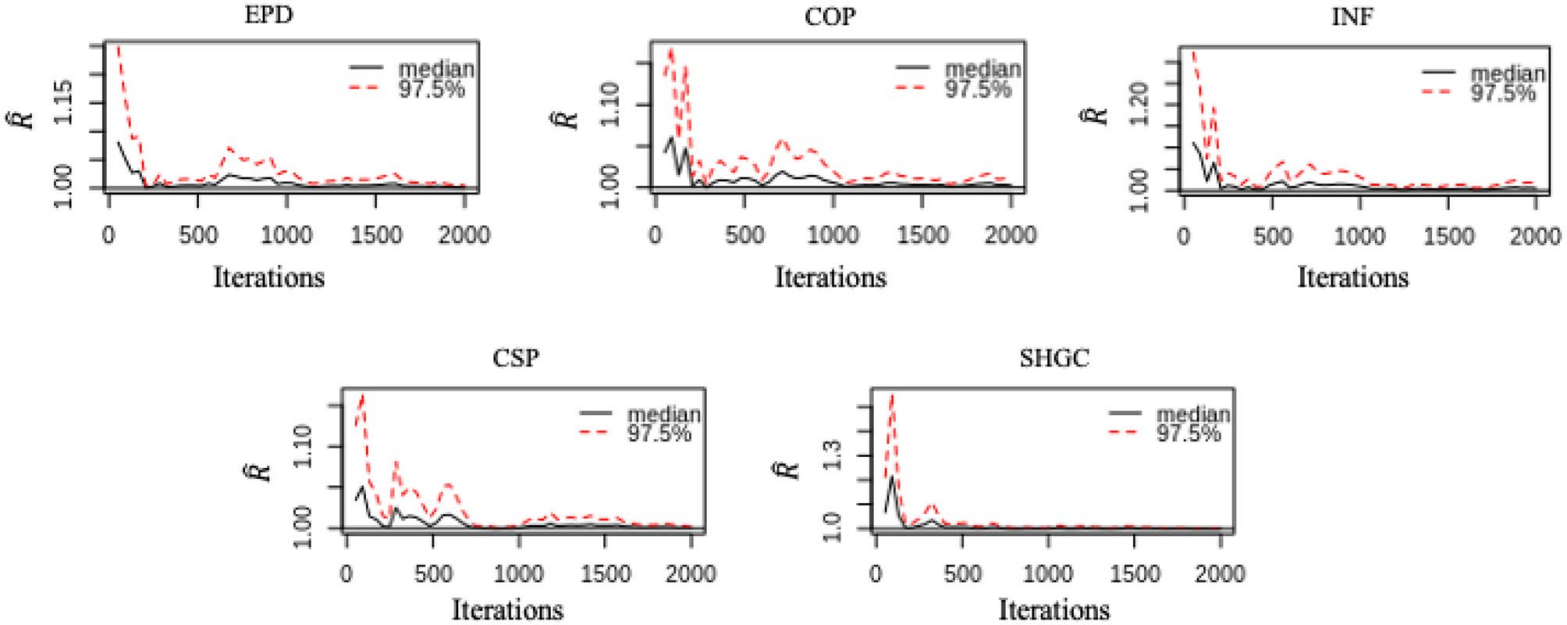
Gelman-Rubin evolution of calibration parameters

The Gelman-Rubin $\widehat{R}$ evaluates the MCMC convergence by comparing the estimated between-chains and within-chain variances for each model parameter. Large Gelman-Rubin $\widehat{R}$ values indicate a divergence. For a converged posterior distribution, $\widehat{R}$ should be smaller than 1.1. The evolution of $\widehat{R}$ of each parameter is shown in Fig. 9. The $\widehat{R}$ values of the parameters are between 1.00 and 1.06. The results of the trace plot and $\widehat{R}$ values demonstrate that the iterations are convergent and all samples from the posterior distributions.

##### Parameter estimation

**Fig. 10:**
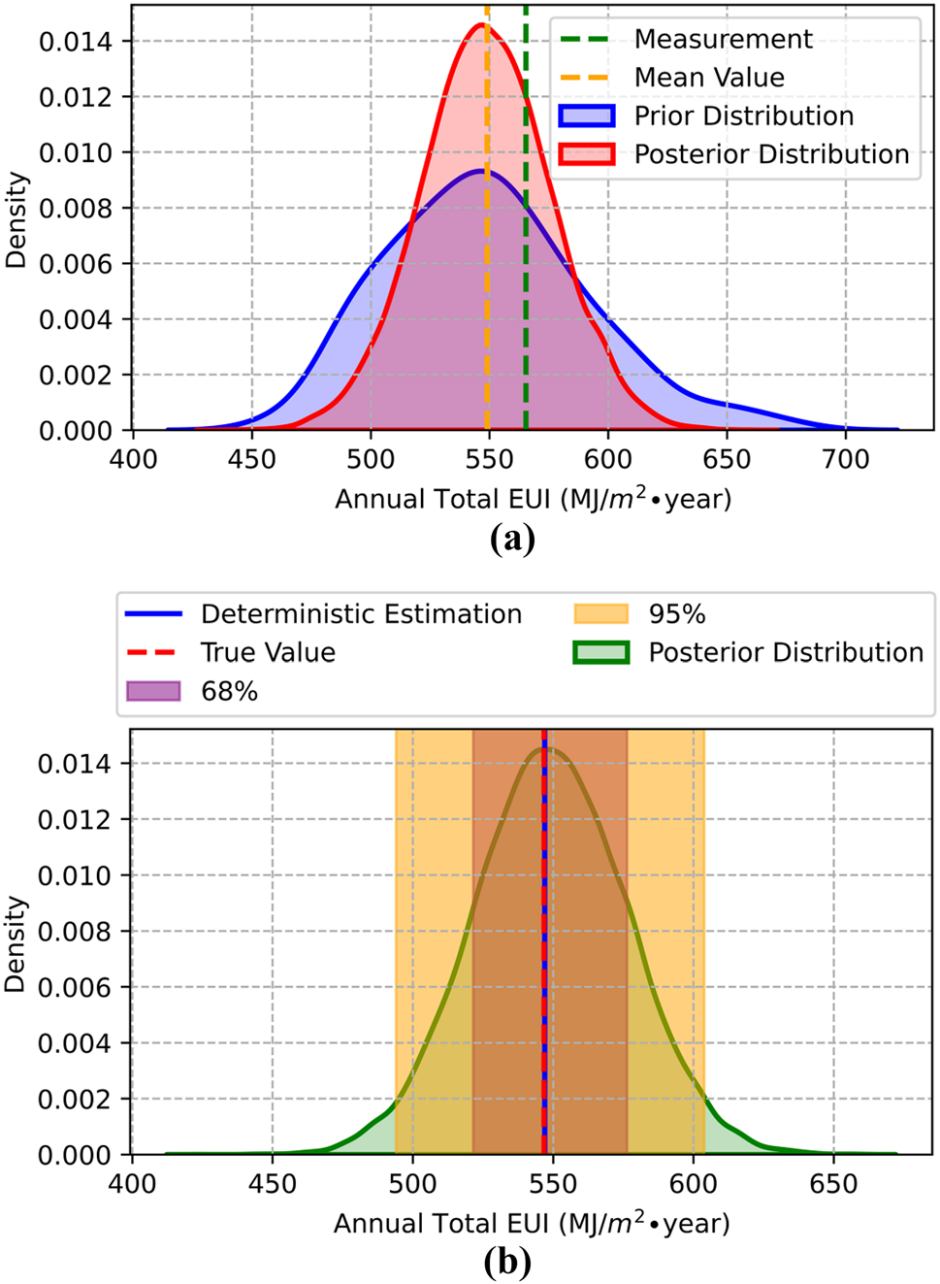
Distributions of annual total EUI: **a** calibration using 2018 weather data, **b** validation using 2019 weather data

The calibrated distribution of the annual total EUI is shown in Fig. 10a. The dotted green line shows the measured annual total EUI of the target building, which is 565.4 MJ/m^2^ year. The blue area is the annual total EUI distribution sampled from prior distributions of calibration model parameters. Its 68% and 95% confidence intervals are (507.0, 590.5) and (465.2, 632.3), respectively. The dotted orange line is the mean value (549.0 MJ/m^2^ year) of the posterior annual total EUI sampled using the posterior distributions of the calibration model parameters represented in the red area. The confidence interval of 68% and 95% of the posterior distribution of the annual total EUI are (521.8, 576.2) and (495.4, 602.6). After the Bayesian Inference calibration, the standard deviation decreased to 65.3% from 41.8 to 27.3. The error rate of the measurement and the mean value of the posterior distribution of the annual total EUI is 2.9%.

**Fig. 11:**
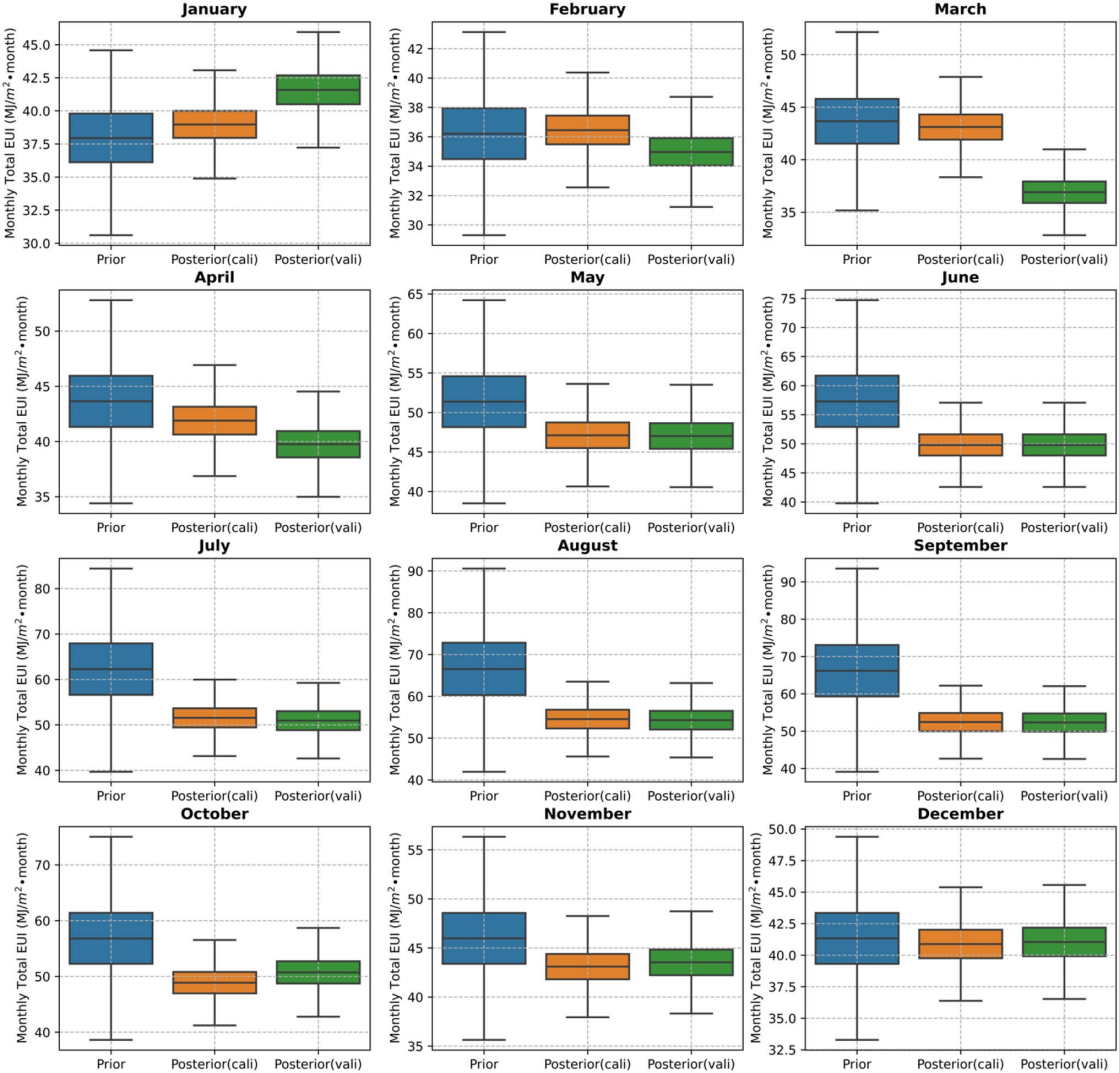
Comparison of stochastically monthly EUIs based on prior/posterior distributions of calibrated model parameters. Posterior (cali)/Posterior(vali) means results generated based on 2018/2019 weather conditions and posterior distributions of calibrated model parameters

The validation results are shown in Fig. 10b. The measurement of annual total EUI by the red dot line is 546.8 MJ/m^2^ $\bullet$ year, the mean value of its posterior estimation in the blue line is 547.0 MJ/m^2^ $\bullet$ year, and the error rate is 0.04%. The validated posterior distribution of annual total EUI is shown in the green area, and its confidence interval of 68% and 95%, colored purple and orange, are (521.4, 576.1) and (494.9, 602.7), respectively. A comparison of stochastically monthly EUIs for calibration and validation based on prior/posterior distributions of calibrated model parameters is shown in Fig. 11.

The CVRMSE values of the monthly total EUI are illustrated in Fig. 16. The calibration CVRMSE is 0.6%, and the validation CVRMSE is 0.5%, which are reasonable considering the monthly calibration tolerance of 15% required by the ASHRAE Guideline 14 [65] and FEMP [66].

The distributions with key values of five unknown parameters are shown in Fig. 15: the CPN is 5. The details in Table 5 show that the error for the COP and CSP is 2.2% and 0.9%, respectively. For the parameter of EPD and INF, the errors are 9.3% and 9.0%, respectively, which may be caused by a broader range. The window SHGC error is 4.2%.

**Table 5:** Details about calibration parameters

Parameter	True value	Prior distribution	Posterior distribution						
		Range with uniform distribution	Mean value	Standard deviation	Quantiles (%)				
					2.5	25	50	75	97.5
EPD	11.8	11.0–15.0	12.9	1.1	11.1	12.0	12.9	13.9	14.9
COP	4.5	3.3–6.0	4.6	0.8	3.4	4.0	4.6	5.3	5.9
INF	1.1E−3	0.0–2.0E−3	1.0E−3	5.8E−4	5.7E−5	5.2E−4	1.0E−3	1.5E−3	1.9E−3
CSP	22.7	22.5–28.5	22.5	0.02	22.5	22.5	22.5	22.5	22.6
WSHGC	9.6E−2	0.0–0.2	0.10	0.06	2.5E−3	0.04	0.09	0.15	0.19

## Case 2: a real residential building

In this section, the platform is applied to a real residential building. The details of preparing and running each module of the calibration platform are not included to avoid duplication. The building information, calibration parameters, and results are provided in the following sections.

### Calibration preparation

**Fig. 12:**
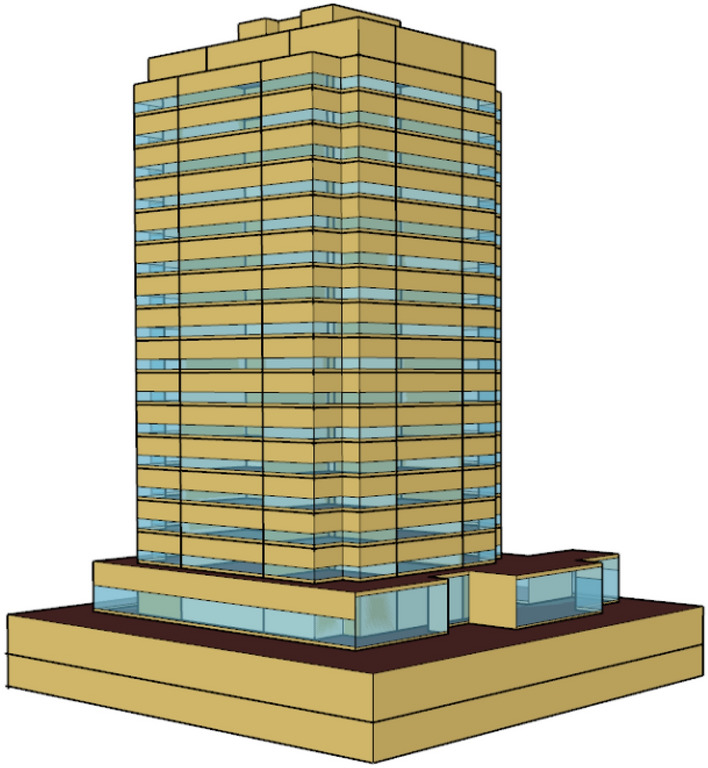
3D rendering model of the real residential building in EnergyPlus

**Fig. 13:**
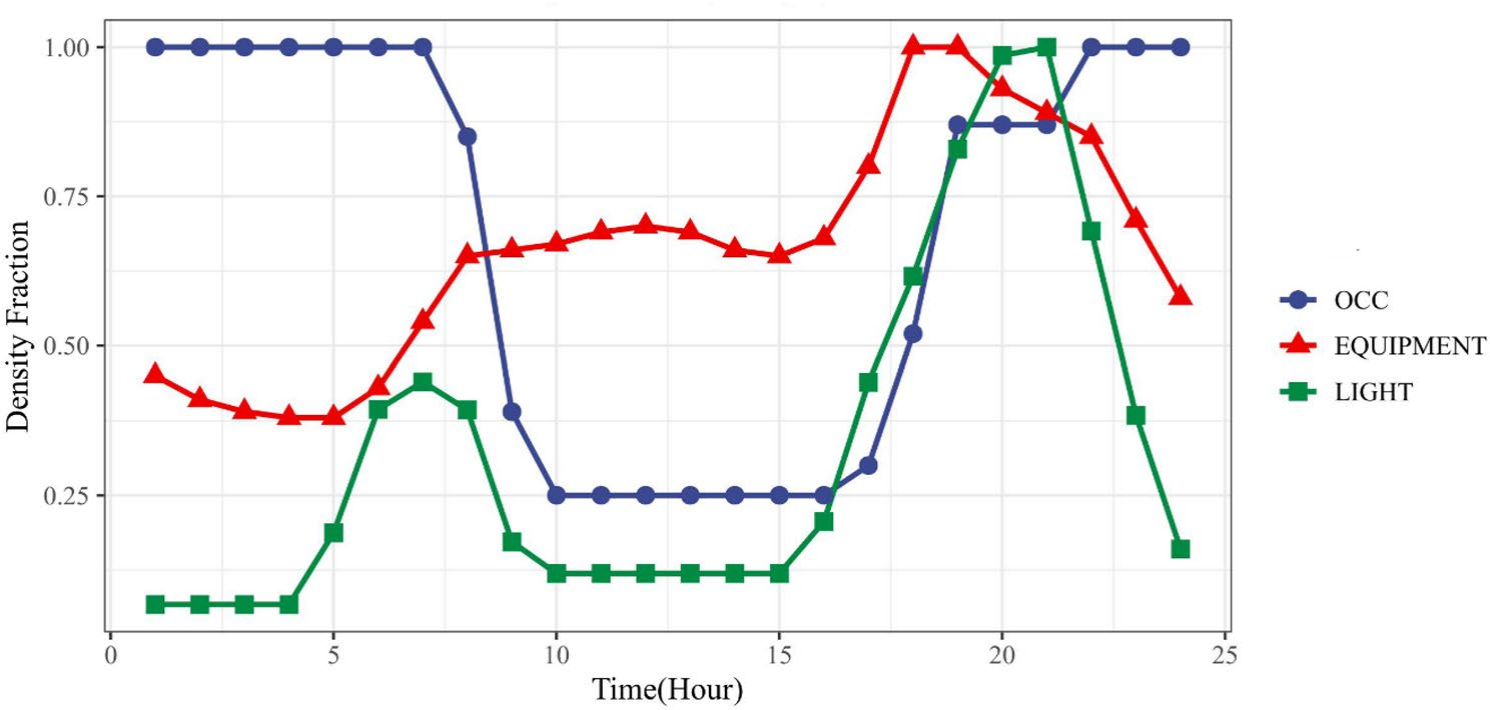
Daily occupancy, lighting, and equipment density fractions

This case study is a real building, Marina Tower (abbreviated to "MT" hereafter), located in Lusail city. The MT is a multi-apartment building with 19 stories (including two basements and a ground floor), as shown in Fig. 12. The total floor area is 26,147.72 m^2^. It is constructed at an orientation of 341.57 $^\circ$ from the North. The two basements are not cooled as they are used for parking. A summary of the building specifications is provided in Table 6. The building cooling energy is provided through a district cooling system. However, it is important to note that this study focuses on building cooling load, and due to building complexity, the HVAC system of the building was not modeled. Instead, the ideal cooling zone method [67] was used to estimate cooling loads. The daily occupancy, equipment, and lighting power density fractions are given in Fig. 13. They are based on ASHRAE 189.1-2009 typical schedules for apartment buildings. In this study, the density fractions were not varied between weekdays and weekends to demonstrate the proposed methods. The same methods would be applicable when the variable density fractions are considered. In fact, according to the information provided by the project stakeholders in Qatar, these constant fractions were reasonable for the study period of interest. The prior distributions of selected calibration parameters are shown in Table 7. The measurements of monthly cooling consumption for the calibration are from August 2020 to July 2021.

**Table 6:** Floor plan summary

Floor	Dimension ($m\times m$)	Floor height (m)	Function	Window-to-wall ratio			
				Front	Rear	Left	Right
2nd basement	60.70 × 58.33	3.9	Parking	/	/	/	/
1st basement	60.70 × 58.33	4.7	Parking	/	/	/	/
Ground floor	46.86 × 44.50	6.1	Lobby	46%	59%	87%	87%
Typical floor (1st–15th)	35.80 × 31.50	3.8	Residential	31%	31%	32%	32%
16th Floor	35.80 × 23.00	4.1	Residential	31%	31%	32%	32%
Roof	21.48 × 11.46	3.8	Pump room & lift lobby	/	/	/	/

**Table 7:** Sensitivity analysis results for the annual energy use intensity of Marina Tower

Parameter	Range of values	Unit	SRC	Random forest	T-Value	SVI	Rank
Cooling setpoint	21–26	$\mathrm{^\circ{\rm C} }$	0.70	165.80	168.74	31.07	1
Equipment power density	2–8	W/m^2^	0.39	102.19	94.47	18.05	2
Ventilation rate	0.0003–0.0006	m^3^/s∙ m^2^	0.36	97.07	85.95	16.75	3
Window SHGC	≤ 0.21	W/m^2^∙K	0.28	49.55	65.36	11.09	4
Lighting power density	3–6	W/m^2^	0.20	24.05	46.66	7.13	5
Infiltration rate	0.1–0.2	ACH	0.14	15.71	32.42	4.88	6
Window U-value	≤ 1.8	W/m^2^∙K	0.10	15.57	22.83	3.74	7
Occupancy density	38–90	m^2^/person	0.09	7.54	20.99	2.98	8
Wall U-value	≤ 0.3	W/m^2^∙K	0.09	3.17	20.62	2.63	9
Roof Insulation U-value	≤ 0.25	W/m^2^∙K	0.02	2.13	3.90	0.60	10
Floor U-value	≤ 0.332	W/m^2^∙K	0.02	0.99	3.94	0.53	11
Solar reflectance of interior diffusing blinds roll	0.4–0.8	/	0.01	0.32	3.22	0.40	12
Window solar transmittance	≤ 0.25	/	0.01	0.23	1.23	0.16	13

### Results

**Fig. 14:**
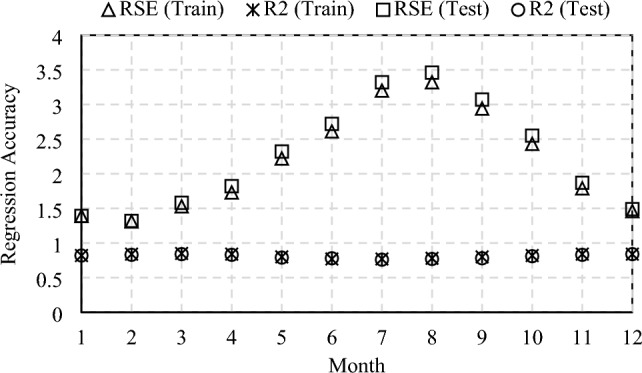
Variation of regression accuracy by month for Case 2

Table 7 summarizes the prior ranges with uniform distribution of model parameters included in the sensitivity analysis process. These parameter ranges were determined according to local or international building codes/standards [58, 68]. The cooling setpoint appears to be the dominant parameter. Floor U-value, solar reflectance of interior diffusing blinds roll, and window solar transmittance are the least influential parameters, which are, therefore, excluded from further simulation. The first five important parameters are selected to use as independent variables in the MLR model and to be calibrated. Figure 14 shows the accuracy of the MLR Meta-model indicated with R^2^ and RES. By employing the developed MLR models in the MCMC process, the details of the posterior distributions of five calibration parameters are presented in Table 8.

**Table 8:** Posterior distributions of calibration parameters

Parameter	Mean value	Standard deviation	Quantiles (%)				
			2.5	25	50	75	97.5
CSP	22.3	1.1	21.06	21.3	22.1	22.9	24.98
EPD	6.23	1.07	4.71	5.17	5.99	7.36	7.85
VEN	5.00E−4	5.70E−5	3.95E−4	4.80E−4	5.20E−4	5.80E−4	5.93E−3
SHGC	0.11	0.06	0.01	0.05	0.11	0.17	0.19
LPD	4.94	1.1	3.03	4.20	5.55	5.74	5.95

**Fig. 15:**
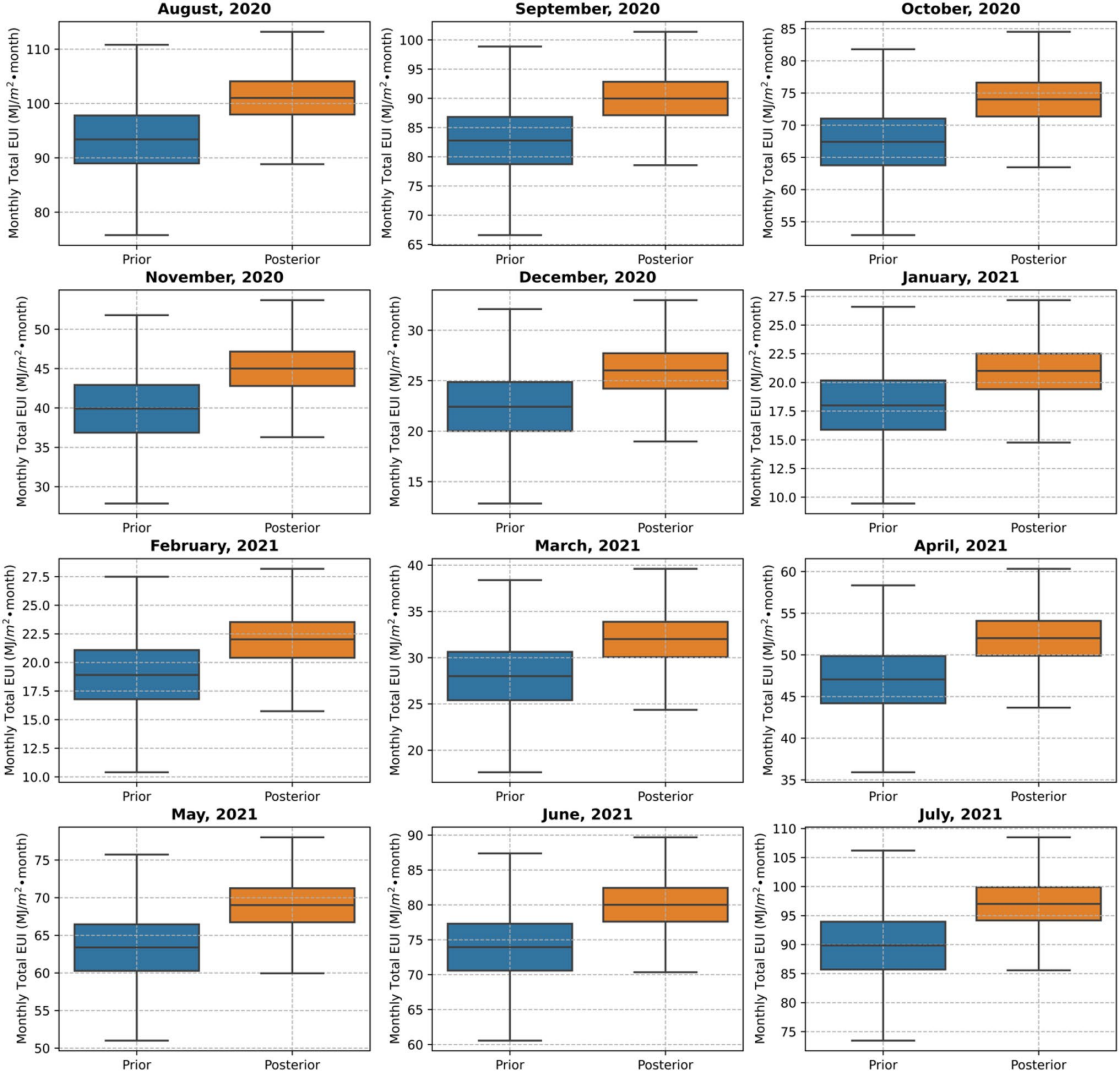
Comparison of stochastically monthly cooling energy use intensity based on prior and posterior distributions of calibrated model parameters

**Fig. 16:**
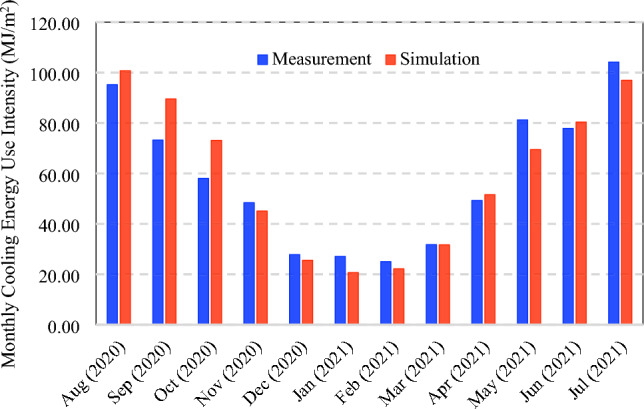
Deterministic monthly cooling energy use intensity between simulation and measurement data

The stochastically monthly cooling energy consumption based on prior and posterior distributions is shown in Fig. 15. Besides that, the comparison between the simulated and measured monthly cooling consumption intensity is shown in Fig. 16, in which the mean values of the posterior distribution of calibration model parameters are used to generate the deterministic results. The calculated CVRMSE is 13.95%, within the acceptable range of 15% based on the ASHRAE Guideline 14 and FEMP [65, 66]. Compared to the previous case, the CVRMSE is higher because the measurements were collected during the COVID-19 pandemic with unrepresentative occupancy profiles and schedules during pre-pandemic. The measured occupancy schedule and profile were not available due to privacy concerns. The current accuracy is considered to be acceptable for demonstration purposes but could be improved further by more measurement data.

## Discussion

**Fig. 17:**
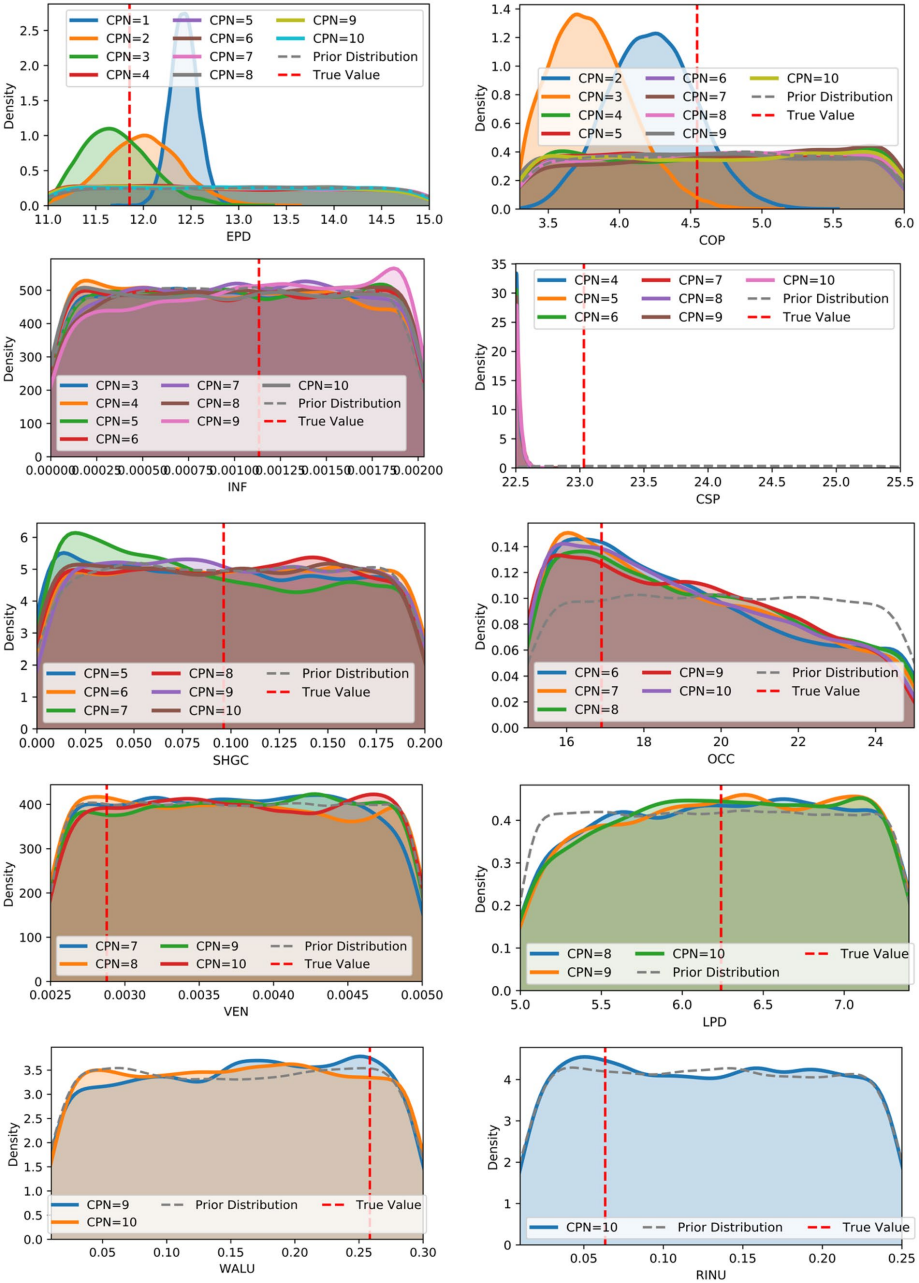
Distribution of calibrated parameters by selecting different calibrated parameter numbers. CPN is the Calibrated Parameter Number

**Fig. 18:**
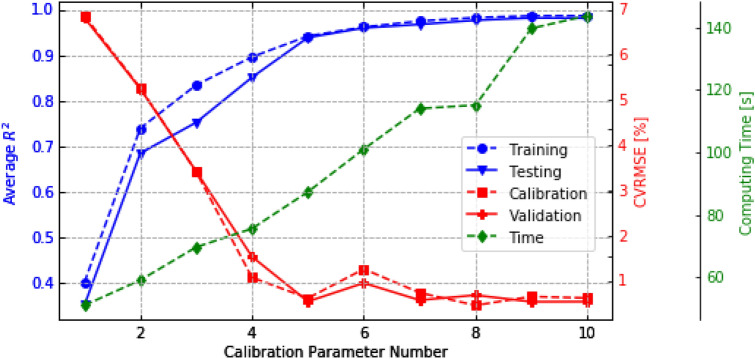
Comparison of Meta-model accuracy, calibration, validation performance, and computing time using different calibration parameter numbers

In the previous sections, five calibration parameters were selected for Bayesian Inference. This section presents a thorough examination of the impact of the number of calibration parameters on the results of the Bayesian Inference calibration process using Case 1. This examination aims to enrich readers' understanding of the implementation of Bayesian inference in the context of building energy applications. Tian [63] stated that the calibration parameters should be less than ten for the Bayesian Inference calibration using MLR. Therefore, 10 cases were conducted with varying the number of calibration parameters from 1 to 10. For example, in case 3, three parameters with the most important impact are selected, namely EPD, COP, and INF, to repeat the procedure of Bayesian Inference calibration. The weather data used for BEM calibration and validation and the selected sampler whose outputs are regarded as measurements maintained as constant in all 10 cases. The posterior distributions of the calibration parameters are shown in Fig. 17. It shows that when the calibration parameter number is greater than 3, over-parameterization occurs, which means the calibration parameters are over the calibration capability. This finding is consistent with the previous study [69], in which a similar conclusion was drawn according to the ratio of the likelihood confidence interval to the prior range. While in Chong and Menberg’s study [37], over-parameterization occurs when the calibration parameter number is greater than 4. The comprehensive comparison, including MLR accuracy (shown by R^2^), calibration and validation accuracy (shown by CVRMSE), and computing time, was demonstrated in Fig. 18. Please note that the Gelman-Rubin values of all cases are within 1.07, which means that the MCMC iterations are convergent for all cases.

The relationship between the number of calibration parameters and the accuracy of the MLR Meta-model shows a logarithmic pattern. When the number is less than 5, the average R^2^ increases dramatically with the calibration parameter. After 5, R^2^ reduces and becomes negligible after 8. Although the MLR model accuracy of the training set is slightly higher than the testing set, the trend will disappear with increasing calibration parameter number. Besides, the number of calibration parameters highly affected the calibration and validation performance estimated by CVRMSE. When the selected calibration parameter is less than 5, the accuracy of calibration and validation increases drastically. However, when the calibration parameter number is greater than 5, the performance becomes stable. The Meta-model's accuracy can explain this observation. In MCMC, the monthly EUI computed by the MLR model was compared to the observations, searching for the optimal posterior distribution of the calibration parameter. If the Meta-model's accuracy is too low, the MCMC inference capability is limited to align the simulation data with the measurements. When the accuracy of the Meta-model is sufficient, increasing calibration parameter numbers lead to higher calibration and validation performance. However, this impact may be negligible. Finally, the computing time of the MCMC process gets longer with the increase of calibrated parameter number. The optimal calibration parameter number should be close to the intersection of CVRMSE performance and computing time with a high Meta-model accuracy and avoiding over-parameterization. Also, the optimal number should be chosen after the best results are shown. Therefore, the optimal calibration parameter number is identified to be 5 in this case.

## Conclusions and future directions

This paper proposed a new platform for building energy model calibration based on Bayesian Inference. The platform was developed using the R language and provided a complete package of the programming environment for a systematic calibration process considering uncertainty. The parameters to be calibrated can be selected from the results of the sensitivity analysis module or defined by the user. The embedded Meta-model development module can reduce computing time by replacing the original BEM during the MCMC process. The developed Meta-model can be applied to future analysis when numerous simulations are needed. The demonstration cases, including a synthetic office building and a real residential building, show the proposed platform's ability to generate results with high estimation accuracy by meeting the calibration requirements of ASHRAE Guideline 14 [65] and FEMP [66]. Besides, the calibration results are expressed in terms of uncertainty and probability. Further, it was found that when the number of calibration parameters is over 5, the calibration and validation performance improves slightly. In contrast, the increase in computing time is almost linear for the MCMC process.

In future research, the proposed platform can be adapted to various other studies, such as building thermal performance assessment and air quality analysis. Besides that, the calibration at a room level is also worth exploring. Currently, only the EnergyPlus model can be coupled with the developed platform, but other BEM models could be integrated too. The monthly data used in the case studies for demonstration is considered a limitation of this study as this data may not be sufficient for the energy performance estimation of large buildings, as noted by Sun and Reddy [38]. Therefore, high-resolution data can be applied to investigate its impact on calibration performance and computational cost. The proposed platform should also be tested further for its functionality, scalability, and robustness. Our research group is currently in the process of releasing the platform publicly to solicit more tests in the future.

## Data Availability

The data that support the findings of this study are available on request from the corresponding author [LW]. The data are not publicly available at the moment due to the undecided data sharing issues with the sponsor for this ongoing project.

## Code availability

The code that supports the findings of this study is available on request from the corresponding author [LW].
